# Anti-Inflammatory Gene Therapy Improves Spatial Memory Performance in a Mouse Model of Alzheimer’s Disease

**DOI:** 10.3233/JAD-215270

**Published:** 2022-02-01

**Authors:** Tai June Yoo

**Affiliations:** a Korea Allergy Clinic, KangNam Gu, Seoul, South Korea; b University of Tennessee Health Science Center, Memphis, TN, USA

**Keywords:** Alzheimer’s disease, amyloid-β protein precursor, genetic therapy, immunotherapy, interleukin-10, interleukin-4, neuroinflammation, spatial memory, transgenic mice, transforming growth factor beta

## Abstract

The immune system plays a critical role in neurodegenerative processes involved in Alzheimer’s disease (AD). In this study, a gene-based immunotherapeutic method examined the effects of anti-inflammatory cellular immune response elements (CIREs) in the amyloid-β protein precursor (AβPP) mouse model. Bi-monthly intramuscular administration, beginning at either 4 or 6 months, and examined at 7.5 through 16 months, with plasmids encoding Interleukin (IL)-10, IL-4, TGF-β polynucleotides, or a combination thereof, into AβPP mice improved spatial memory performance. This work demonstrates an efficient gene therapy strategy to downregulate neuroinflammation, and possibly prevent or delay cognitive decline in AD.

## INTRODUCTION

Alzheimer’s disease (AD), the most common form of dementia, is not a normal part of aging, but rather a chronic neurodegenerative pathology associated with neuroinflammation, extracellular amyloid-β (Aβ) plaques, and hyperphosphorylated tau, which leads to progressive cognitive decline in older adults [1–3]. The specific cause of AD remains unclear, but it may collectively involve the accumulation of activated microglia, astrocytes, and proliferative T cells which target extracellular filamentous abnormal Aβ protein deposits in the brain [4–7]. Although most neurodegenerative diseases are not classically considered autoimmune, in some instances, chronic neuroinflammation in aging can exacerbate a progressively declining innate immune system leading to further neuronal damage [8, 9].

The adaptive immune system can be broadly classified into two types of inflammatory activity: cellular and humoral (antibody). Among cytokine responses, proinflammatory T helper type 1 (Th1) (i.e., interferon-gamma (IFN-γ), tumor necrosis factor (TNF-α), IL-1, IL-2, IL-12), an anti-inflammatory Th2 (i.e., IL-4, IL-5, IL-10, IL-13), Th3 (TGF-β), and Th17 are involved in neurodegenerative disease and could be targeted for therapy. Moreover, anti-inflammatory CIREs such as IL-10 and TGF-β play a critical role in neurodegenerative autoimmune diseases such as multiple sclerosis, Parkinson’s disease, and amyotrophic lateral sclerosis [10–14]. Although immunotherapeutic clinical trials were previously halted, emerging work continues to corroborate the importance of T cell recognition and autoimmune susceptibility in the etiology of AD [15–17].

For patients experiencing mild cognitive impairment, an elevated presence of proinflammatory TNF-α concurrent with decreased anti-inflammatory TGF-β levels was observed, resulting in a greater risk conversion towards AD [18]. Similarly, TNF-α and other proinflammatory cytokines such as IL-1β and IL-6 were reported to impact anti-inflammatory processes and increase amyloid brain deposition in transgenic AD mice [19–21], with the latter cytokine driving blood-brain barrier dysfunction [22]. Anti-inflammatory cytokines, IL-4 and IL-10, have also garnered therapeutic interest due to their immunomodulatory role in the autoreactive T cell repertoire of neurodegenerative diseases [23–25]. Researchers evaluating IL-4 and IL-10 polymorphisms in patient populations have reported that a relative paucity of the genetic disruptions in these Th2 cytokines increases susceptibility to developing AD [26–31]. Therefore, the specific role these and other anti-inflammatory cytokines play in preventing or ameliorating neurodegeneration in AD not only warrants further investigation, but also requires new experimental approaches.

For example, gene-based technologies could provide a promising therapeutic strategy to ameliorate neurodegenerative disease due to administration ease, an efficacious and safe profile, and long-lasting effects [32–35]. Previous Yoo laboratory gene transfer work, examining anti-inflammatory response using a clinically relevant allergen to induce experimental autoimmune hearing loss, was successful in controlling autoimmune reaction severity through suppression of Th1-type proinflammatory responses and inducing IL-10-secreting regulatory T cells [36]. This non-toxic naked DNA delivery technique suggests exogenous IL-10 could restore immunological homeostasis by suppressing the autoimmune response and generate an endogenous regulatory IL-10 profile. Since chronic inflammation appears to trigger T cell-mediated autoimmune disease, the present study assessed whether anti-inflammatory CIRE gene therapy could also improve spatial memory performance in the amyloid-β protein precursor (*AβPP*) mouse, and thus prevent or delay AD onset.

## MATERIALS AND METHODS

### Transgenic animals

Transgenic mice (Tg-2576) containing the K670N/M671L (AβPP) Swedish double mutation, which leads to familial early onset AD [37], were obtained from The Jackson Laboratory (Bar Harbor, ME, U.S.A.) and maintained at the University of Tennessee, Memphis animal facility after experimental approval by the Institutional Animal Care and Use Committee of the University of Tennessee.

### Plasmid DNA preparation and transfection reagents

Polynucleotide constructs under a simian virus 40 promoter encoding a CIRE were used: IL-4, IL-10, TGF-β (i.e., GenBank Accession No. M13982, 55 SEQ ID NO:12), (M57627, SEQ ID NO:14), (M60316, SEQ ID NO:16), respectively. Expression of CIRE naked DNA plasmids utilized the pVAX1 vector (Invitrogen, Carlsbad, CA) and cytomegalovirus promoter/enhancer sequences. A control vector without the CIRE genes was developed by digesting related plasmid DNA with *Eco*RI, followed by ligating the agarose gel-purified vector fragment. Large-scale purification of all plasmid DNA was conducted with Endo Free Plasmid Maxi kits (Qiagen, Valencia, CA). Methodology was reported [38–42], with dosage effective at least 1–5 weeks after injection, and adopted from previous studies [36, 43]. Male and female AβPP mice were bi-monthly, intramuscularly injected with either 100μg of a blank vector in 100μl of phospate-buffered saline for control or the same amount of naked DNA encoding CIREs. Mice were used or maintained until age 60 weeks, then sacrificed, brains removed, snap-frozen in liquid nitrogen, and stored at -80°C.

### Spatial memory performance evaluation in untreated and treated AβPP mice

**Fig. 1:**
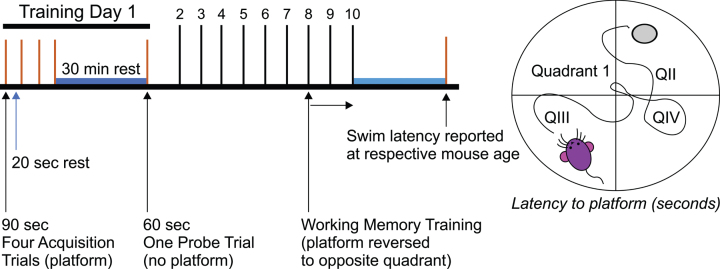
Evaluation of spatial memory in AβPP mice using the Morris Water Maze. The MWM is an intermittently sensitive test to evaluate reference memory performance for AβPP mice [47].

Spatial learning and memory were assessed using the Morris Water Maze task [44] in a circular tank (80 cm wide, 80 cm deep) with a non-distinct submerged central platform (15 cm wide, 1 cm below opaque water, 23°C) (Fig. 1). All trials were recorded with an overhead camera. Maximum swim time for each trial was 90 s followed by a 20-s platform rest. Each mouse was trained for five days, four trials per day with randomized starting points. Probe trials were performed without the platform 30 min after the last trial. Mice were released opposite the target quadrant and allowed 60 s to swim. Following retraining (day 7), the platform was moved to the opposite quadrant for reversal training (days 8–10). A retention test was conducted 30 min after the last acquisition trial, and latency (seconds to platform) was registered (Table 1). Combined cytokine-treated animals were only tested at 13 and 16 months of age.

**Table 1:** Morris Water Maze results of 7.5 through 16 months with bi-monthly DNA-based immunotherapy beginning either at 4 or 6 months of age in AβPP mice

Testing Age (month)	7.5	8	9	9.5	11	13	16
Normal Untreated Mouse	6	6	6	d.n.r	d.n.r	6	6
AβPP control vector	120	120	160, 52	144, 52	144	220, 100	62
AβPP + TGF-β	23	23	80, 70	72, 66	78, 6	80, 30	14
AβPP + IL-10	15	15	8, 7, 15	8, 7	2, 7	1, 1, 8	3, 4
AβPP + IL-4	d.n.r	d.n.r	10	10	10	10	4
AβPP + (IL-10 + IL-4)	d.n.r	d.n.r	d.n.r	d.n.r	d.n.r	1, 2	3
AβPP + (IL-10 + TGF-β)	d.n.r	d.n.r	d.n.r	d.n.r	d.n.r	45, 44	d.n.r

Normal Untreated Mice (*n* = 5), AβPP control vector (*n* = 10), TGF-β (*n* = 11), IL-10 (*n* = 14), IL-4 (*n* = 5), IL-10 + IL-4 (*n* = 3), and IL-10 + TGF-β (*n* = 2) latencies for individual mice were registered and indicated by numerical values. d.n.r., data not recorded.

### Data analysis

Latency times with animals (*n*≥7) administered with CIREs beginning at 6 months were measured at specific ages (7.5, 9.5, 11, 13, 16 months) and analyzed by Welch’s *t*-test and one-way ANOVA with Tukey HSD *Post Hoc* in *R open source software* (https://cran.case.edu/) [45]. Threshold values of *p* = 0.05 were considered statistically significant.

## RESULTS

### Early and late TGF-β, IL-10, or IL-4 gene therapy prevent and ameliorate AβPP mice memory deficits

**Fig. 2:**
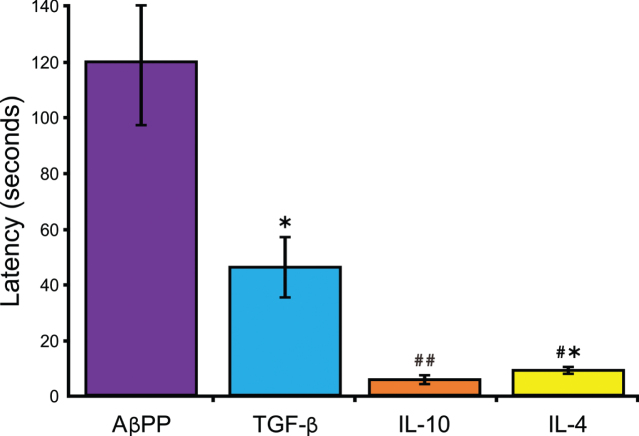
AβPP mice treated with TGF-β, IL-10, or IL-4 improves spatial memory. AβPP mice injected bi-monthly, beginning at 6 months, with either TGF-β (*n* = 8) or IL-10 (*n* = 10) and tested at 7.5, 9.5, 11, 13, and 16 months showed significant difference compared to AβPP mice receiving control vector (*n* = 7) [*F(*2,22) = 22.69*, p* < 0.001)]. AβPP mice administered IL-4 (*n* = 4) also reduced swim latency compared to TGF-β (*p* = 0.011). Mean±S.E.M., Welch’s *t* test; ^*^p < 0.05, ^#^*p* < 0.01.

Mice injected bi-monthly with naked DNA encoding TGF-β, IL-10, or IL-4 beginning at 4-months of age (8 & 9 months columns), when hippocampal lesions begin to appear in the AβPP model, and tested in the MWM at 8 or 9 months (when lesions fully form [46]) reduced latency times to platform compared to age-matched AβPP mice receiving a control blank vector (Table 1). Interestingly, AβPP mice receiving either TGF-β, IL-10, or IL-4, or a CIRE gene combination bi-monthly beginning at 6 months, and examined at 7.5 through 16 months, also reduced latency-to-platform behavior. Overall, AβPP mice administered TGF-β, IL-10, or IL-4 naked DNA performed significantly better compared to AβPP controls (*p* = 0.014, 0.002, and 0.002, respectively; Fig. 2). No significant difference was observed between Normal Untreated (not illustrated) and IL-10 or IL-4 treated mice (*p* = 0.777, 0.194, respectively).

## DISCUSSION

The present study demonstrates that gene-based immunotherapy, with pleiotropic anti-inflammatory cytokines IL-10, IL-4, or TGF-β improves spatial memory performance in a mouse model of AD. AβPP mice treated bi-monthly, beginning at age 4 or 6 months, with a single CIRE dose (or a combination thereof, in older animals) (Table 1), rescued an AD-associated behavioral phenotype. Although AD mouse models, including over 300 therapeutic investigations with the Tg-2576 line [48], presented pre-clinical limitations which resulted in untoward clinical trial outcomes [49–51], nevertheless still comprise ∼45% of AD drug development and continue to provide insight on temporal cell signaling in neurodegenerative disease [52, 53].

For instance, hippocampal IL-10 or IL-4 overexpression through an adeno-associated virus (AAV) in amyloid precursor protein+presenilin-1 bigenic mice increases neurogenesis and improves cognition without affecting hippocampal beta-amyloidosis [54, 55]. A separate group also reported that AAV-IL-4 induced an anti-inflammatory response from an alternative activated macrophage phenotype while stimulating microglia and astrogliosis [56]. However, in two different AD models, IL-10- and IL-4-AAV modification increased hippocampal and cortical Aβ accumulation and impaired memory, resulting in aberrant innate immune amyloidosis [57–59]. While viral vector technology for neuronal system delivery advances, introducing exogenous Th2 cytokine into a chronically inflamed system may unintentionally exacerbate and accelerate neuropathology [60–63]. Precise therapeutic intervention may be required. As an example, non-viral immunotherapy for AD could utilize the appropriate biomolecule, adjuvant and dose based on the patient’s metabolic and stratified risk profile [64, 65].

Although anti-inflammatory gene therapy, in the present report, improved overall spatial memory deficits in AβPP mice, it remains unclear whether the corresponding cytokine levels increased in the periphery and/or neuronal tissue with the given dose. Previous experiments in the Yoo laboratory confirmed, through harvested splenocyte cultures and cochlear histology of IL-10^–/–^ mice with experimental autoimmune hearing loss, that intramuscular injection of 100μg IL-10 DNA provided sufficient peripheral and cranial IL-10 production [36]. Interestingly, proinflammatory-induced neurodegeneration in an AD rat model was also alleviated in a dose-dependent manner with TGF-β [66]. Notably, combinatorial TGF-β/IL-10 plasmid DNA immunotherapy has already been explored to treat humoral autoimmune diseases [67]. Safe, novel, and optimized gene-based neuroimmunotherapies will be essential as drug development advances [68–70].

In oncology, ‘cytokine synergy’ infers that combined therapeutic potency is greater than any of the individual cytokines alone [71]. Intriguingly, older mice administered IL-10 + IL-4, and to a lesser extent IL-10 + TGF-β, could augment spatial memory in AβPP mice (Table 1), suggesting synergistic anti-inflammatory AD amelioration. Additionally, plasmid delivery of DNA encoding IL-10/IL-4 prevents autoimmune diabetes in nonobese diabetic mice [72], while fusion protein treatments with these Th2 cytokines alleviates inflammatory pain [73, 74]. Currently, inflammation in these concomitant diseases exacerbates cognitive decline [75–79], but synergistic cytokine gene therapy could provide health benefits not only for the aging population, but society as a whole.

Finally, AD mouse models exhibit anxiety, age/gender performance variability, elevated retinal Aβ, increased proinflammatory Th1 cytokines, and early-onset biomarker absence, which may confound behavioral data [53, 80–85]. Despite their putatively dubious nature, gene-based immunotherapy research in transgenic mice should continue exploring enhanced delivery systems and promoters, complementing adjuvants, and confirmation of experimental results in other AD models, such as rabbits, where artificially-induced risks factors are closer to human AD [86–90]. Furthermore, clinical trials for neurodegenerative disease involving naked DNA require more investment due to plasmid DNA biocompatibility, lower manufacturing cost, efficient production, and storage stability [91–93]. In summary, the present study provides an efficient strategy of preventing/delaying AD onset through down regulation of chronic inflammation using gene therapy.

## References

[1] Heneka MT , Carson MJ , El Khoury J , Landreth GE , Brosseron F , Feinstein DL , Jacobs AH , Wyss-Coray T , Vitorica J , Ransohoff RM , Herrup K , Frautschy SA , Finsen B , Brown GC , Verkhratsky A , Yamanaka K , Koistinaho J , Latz E , Halle A , Petzold GC , Town T , Morgan D , Shinohara ML , Perry VH , Holmes C , Bazan NG , Brooks DJ , Hunot S , Joseph B , Deigendesch N , Garaschuk O , Boddeke E , Dinarello CA , Breitner JC , Cole GM , Golenbock DT , Kummer MP (2015) Neuroinflammation in Alzheimer’s disease. Lancet Neurol 14, 388–405. 25792098 10.1016/S1474-4422(15)70016-5 PMC5909703

[2] Selkoe DJ , Hardy J (2016) The amyloid hypothesis of Alzheimer’sdisease at 25 years. EMBO Mol Med 8, 595–608. 27025652 10.15252/emmm.201606210 PMC4888851

[3] Long JM , Holtzman DM (2019) Alzheimer disease: An update on pathobiology and treatment strategies. Cell 179, 312–339. 31564456 10.1016/j.cell.2019.09.001 PMC6778042

[4] Monsonego A , Zota V , Karni A , Krieger JI , Bar-Or A , Bitan G , Budson AE , Sperling R , Selkoe DJ , Weiner HL (2003) Increased T cell reactivity to amyloid beta protein in older humans and patients with Alzheimer disease. J Clin Invest 112, 415–422. 12897209 10.1172/JCI18104 PMC166296

[5] Fisher Y , Nemirovsky A , Baron R , Monsonego A (2010) T cells specifically targeted to amyloid plaques enhance plaque clearance in a mouse model of Alzheimer’s disease. PLoS One 5, e10830. 20520819 10.1371/journal.pone.0010830 PMC2877087

[6] Orre M , Kamphuis W , Osborn LM , Jansen A , Kooijman L , Bossers K , Hol EM (2014) Isolation of glia from Alzheimer’s mice revealsinflammation and dysfunction. Neurobiol Aging 35, 2746–2760. 25002035 10.1016/j.neurobiolaging.2014.06.004

[7] Fakhoury M (2018) Microglia and astrocytes in Alzheimer’s disease: Implications for therapy. Curr Neuropharmacol 16, 508–518. 28730967 10.2174/1570159X15666170720095240 PMC5997862

[8] Amor S , Peferoen LA , Vogel DY , Breur M , van der Valk P , Baker D , van Noort JM (2014) Inflammation in neurodegenerative diseases–an update. Immunology 142, 151–166. 24329535 10.1111/imm.12233 PMC4008224

[9] Guzman-Martinez L , Maccioni RB , Andrade V , Navarrete LP , Pastor MG , Ramos-Escobar N (2019) Neuroinflammation as a common feature of neurodegenerative disorders. Front Pharmacol 10, 1008. 31572186 10.3389/fphar.2019.01008 PMC6751310

[10] Martinez Doncel A , Rubio A , Arroyo R , de las Heras V , Martín C , Fernandez-Arquero M , de la Concha EG (2002) Interleukin-10 polymorphisms in Spanish multiple sclerosis patients. J Neuroimmunol 131, 168–172. 12458048 10.1016/s0165-5728(02)00248-5

[11] Li D , Song X , Huang H , Huang H , Ye Z (2018) Association of Parkinson’s disease-related pain with plasma interleukin-1, interleukin-6, interleukin-10, and tumour necrosis factor-α. Neurosci Lett 683, 181–184. 30063943 10.1016/j.neulet.2018.07.027

[12] Porro C , Cianciulli A , Panaro MA (2020) The regulatory role of IL-10 in neurodegenerative diseases. Biomolecules 10, 1017. 10.3390/biom10071017 PMC7407888 32659950

[13] Strickland MR , Ibanez KR , Yaroshenko M , Diaz CC , Borchelt DR , Chakrabarty P (2020) IL-10 based immunomodulation initiated at birth extends lifespan in a familial mouse model of amyotrophic lateral sclerosis. Sci Rep 10, 20862. 33257786 10.1038/s41598-020-77564-3 PMC7705692

[14] Lindestam Arlehamn CS , Dhanwani R , Pham J , Kuan R , Frazier A , Rezende Dutra J , Phillips E , Mallal S , Roederer M , Marder KS , Amara AW , Standaert DG , Goldman JG , Litvan I , Peters B , Sulzer D , Sette A (2020) α-Synuclein-specific T cell reactivity is associated with preclinical and early Parkinson’s disease. Nat Commun 11, 1875. 32313102 10.1038/s41467-020-15626-w PMC7171193

[15] Togo T , Akiyama H , Iseki E , Kondo H , Ikeda K , Kato M , Oda T , Tsuchiya K , Kosaka K (2002) Occurrence of T cells in the brain of Alzheimer’s disease and other neurological diseases. J Neuroimmunol 124, 83–92. 11958825 10.1016/s0165-5728(01)00496-9

[16] Orgogozo JM , Gilman S , Dartigues JF , Laurent B , Puel M , Kirby LC , Jouanny P , Dubois B , Eisner L , Flitman S , Michel BF , Boada M , Frank A , Hock C (2003) Subacute meningoencephalitis in a subset of patients with AD after Abeta42 immunization. Neurology 61, 46–54. 12847155 10.1212/01.wnl.0000073623.84147.a8

[17] Lindestam Arlehamn CS , Garretti F , Sulzer D , Sette A (2019) Roles for the adaptive immune system in Parkinson’s and Alzheimer’s diseases. Curr Opin Immunol 59, 115–120. 31430650 10.1016/j.coi.2019.07.004 PMC6774843

[18] Tarkowski E , Andreasen N , Tarkowski A , Blennow K (2003) Intrathecal inflammation precedes development of Alzheimer’s disease. J Neurol Neurosurg Psychiatry 74, 1200–1205. 12933918 10.1136/jnnp.74.9.1200 PMC1738668

[19] Patel NS , Paris D , Mathura V , Quadros AN , Crawford FC , Mullan MJ (2005) Inflammatory cytokine levels correlate with amyloid load in transgenic mouse models of Alzheimer’s disease. J Neuroinflammation 2, 9. 15762998 10.1186/1742-2094-2-9 PMC555557

[20] Millington C , Sonego S , Karunaweera N , Rangel A , Aldrich-Wright JR , Campbell IL , Gyengesi E , Münch G (2014) Chronic neuroinflammation in Alzheimer’s disease: New perspectives on animal models and promising candidate drugs. Biomed Res Int 2014, 309129. 25025046 10.1155/2014/309129 PMC4083880

[21] Reale M , D’Angelo C , Costantini E , Di Nicola M , Yarla NS , Kamal MA , Salvador N , Perry G (2018) Expression profiling of cytokine, cholinergic markers, and amyloid-β deposition in the APPSWE/PS1dE9 mouse model of Alzheimer’s disease pathology. J Alzheimers Dis 62, 467–476. 29439355 10.3233/JAD-170999 PMC5817902

[22] Riphagen JM , Ramakers IHGM , Freeze WM , Pagen LHG , Hanseeuw BJ , Verbeek MM , Verhey FRJ , Jacobs HIL (2020) Linking APOE-ɛ4, blood-brain barrier dysfunction, and inflammation to Alzheimer’s pathology. Neurobiol Aging 85, 96–103. 31733942 10.1016/j.neurobiolaging.2019.09.020

[23] Anderson AC , Reddy J , Nazareno R , Sobel RA , Nicholson LB , Kuchroo VK (2004) IL-10 plays an important role in the homeostatic regulation of the autoreactive repertoire in naive mice. J Immunol 173, 828–834. 15240669 10.4049/jimmunol.173.2.828

[24] Maynard CL , Weaver CT (2008) Diversity in the contribution of interleukin-10 to T-cell-mediated immune regulation. Immunol Rev 226, 219–233. 19161427 10.1111/j.1600-065X.2008.00711.x PMC2630587

[25] Miró-Mur F , Urra X , Ruiz-Jaén F , Pedragosa J , Chamorro Á , Planas AM (2020) Antigen-dependent T cell responseto neural peptides after human ischemic stroke. Front CellNeurosci 3, 206. 10.3389/fncel.2020.00206 PMC7348665 32719588

[26] Lio D , Licastro F , Scola L , Chiappelli M , Grimaldi LM , Crivello A , Colonna-Romano G , Candore G , Franceschi C , Caruso C (2003) Interleukin-10 promoter polymorphism in sporadic Alzheimer’s disease. Genes Immun 4, 234–238. 12700599 10.1038/sj.gene.6363964

[27] Li W , Qian X , Teng H , Ding Y , Zhang L (2014) Association of interleukin-4 genetic polymorphisms with sporadic Alzheimer’s disease in Chinese Han population. Neurosci Lett 563, 17–21. 24463336 10.1016/j.neulet.2014.01.019

[28] Soosanabadi M , Bayat H , Kamali K , Saliminejad K , Banan M , Khorram Khorshid H (2015) Association study of IL-4 -590C/T and DDX39B -22G/C polymorphisms with the risk of late-onset Alzheimer’s disease in Iranian population. Curr Aging Sci 8, 276–281. 26265379 10.2174/187460980803151027125919

[29] Su F , Bai F , Zhang Z (2016) Inflammatory cytokines and Alzheimer’s disease: A review from the perspective of genetic polymorphisms. Neurosci Bull 32, 469–480. 27568024 10.1007/s12264-016-0055-4 PMC5563762

[30] Mun MJ , Kim JH , Choi JY , Jang WC (2016) Genetic polymorphisms of interleukin genes and the risk of Alzheimer’s disease: An update meta-analysis. Meta Gene 8, 1–10. 27014584 10.1016/j.mgene.2016.01.001 PMC4792847

[31] Babić Leko M , Nikolac Perković M , Klepac N , Štrac DŠ , Borovečki F , Pivac N , Hof PR , Šimić G (2020) IL-1β, IL-6, IL-10, and TNFα single nucleotidepolymorphisms in human influence the susceptibility to Alzheimer’sdisease pathology. J Alzheimers Dis 75, 1029–1047. 32390629 10.3233/JAD-200056

[32] Wolff JA , Ludtke JJ , Acsadi G , Williams P , Jani A (1992) Long-term persistence of plasmid DNA and foreign gene expression in mouse muscle. Hum Mol Genet 1, 363–369. 1301910 10.1093/hmg/1.6.363

[33] Matsumoto Y , Niimi N , Kohyama K (2013) Development of a new DNA vaccine for Alzheimer disease targeting a wide range of aβ species and amyloidogenic peptides. PLoS One 8, e75203. 24086465 10.1371/journal.pone.0075203 PMC3785508

[34] Martins YA , Tsuchida CJ , Antoniassi P , Demarchi IG (2017) Efficacy and safety of the immunization with DNA for Alzheimer’s disease in animal models: A systematic review from literature. J Alzheimers Dis Rep 1, 195–217. 30480238 10.3233/ADR-170025 PMC6159633

[35] Suschak JJ , Williams JA , Schmaljohn CS (2017) Advancements in DNA vaccine vectors, non-mechanical delivery methods, and molecular adjuvants to increase immunogenicity. Hum Vaccin Immunother 13, 2837–2848. 28604157 10.1080/21645515.2017.1330236 PMC5718814

[36] Zhou B , Kermany MH , Cai Q , Cai C , Zhou Y , Nair U , Liu W , Yoo TJ (2012) Experimental autoimmune hearing loss is exacerbated in IL-10-deficient mice and reversed by IL-10 gene transfer. Gene Ther 19, 228–235. 21697956 10.1038/gt.2011.88

[37] Hsiao K , Chapman P , Nilsen S , Eckman C , Harigaya Y , Younkin S , Yang F , Cole G (1996) Correlative memory deficits, Abeta elevation, and amyloid plaques in transgenic mice. Science 274, 99–102. 8810256 10.1126/science.274.5284.99

[38] Wolff JA , Malone RW , Williams P , Chong W , Acsadi G , Jani A , Felgner PL (1990) Direct gene transfer into mouse muscle *in vivo*. Science 247, 1465–1468. 1690918 10.1126/science.1690918

[39] Raz E , Carson DA , Parker SE , Parr TB , Abai AM , Aichinger G , Gromkowski SH , Singh M , Lew D , Yankauckas MA (1994) Intradermal gene immunization: The possible role of DNA uptake in the induction of cellular immunity to viruses. Proc Natl Acad Sci U S A 91, 9519–9523. 7937799 10.1073/pnas.91.20.9519 PMC44844

[40] Sato Y , Roman M , Tighe H , Lee D , Corr M , Nguyen MD , Silverman GJ , Lotz M , Carson DA , Raz E (1996) Immunostimulatory DNA sequences necessary for effective intradermal gene immunization. Science 273, 352–354. 8662521 10.1126/science.273.5273.352

[41] Wivel NA , Wilson JM (1998) Methods of gene delivery. Hematol Oncology Clin North Am 12, 483–501. 10.1016/s0889-8588(05)70004-6 9684094

[42] Yoo TJ (2013) Treatment and prevention of neurodegenerative diseases using gene therapy. United States Patent 8,247,385 B2.

[43] Kwon SS , Kim N , Yoo TJ (2001) The effect of vaccination with DNA encoding murine T-cell epitopes on the Der p 1 and 2 induced immunoglobulin E synthesis. Allergy 56, 741–748. 11488667 10.1034/j.1398-9995.2001.056008741.x

[44] Morris R (1984) Developments of a water-maze procedure for studying spatial learning in the rat. J Neurosci Methods 11, 47–60. 6471907 10.1016/0165-0270(84)90007-4

[45] R Core Team (2013) R: A language and environment for statistical computing. Vienna, Austria: R Foundation for Statistical Computing. Retrieved from http://www.R-project.org (accessed on July 13, 2021).

[46] Drummond E , Wisniewski T (2017) Alzheimer’s disease: Experimental models and reality. Acta Neuropathol 133, 155–175. 28025715 10.1007/s00401-016-1662-x PMC5253109

[47] Stewart S , Cacucci F , Lever C (2011) Which memory task for my mouse? A systematic review of spatial memory performance in the Tg2576 Alzheimer’s mouse model. J Alzheimers Dis 26, 105–126. 10.3233/JAD-2011-101827 21558645

[48] Zahs KR , Ashe KH (2010) ‘Too much good news’ - are Alzheimer mouse models trying to tell us how to prevent, not cure, Alzheimer’s disease? Trends Neurosci 33, 381–389. 20542579 10.1016/j.tins.2010.05.004

[49] Schenk D (2002) Amyloid-beta immunotherapy for Alzheimer’s disease: The end of the beginning. Nat Rev Neurosci 3, 824–828. 12360327 10.1038/nrn938

[50] Hung SY , Fu WM (2017) Drug candidates in clinical trials for Alzheimer’s disease. J Biomed Sci 24, 47. 28720101 10.1186/s12929-017-0355-7 PMC5516350

[51] Sasaguri H , Nilsson P , Hashimoto S , Nagata K , Saito T , De Strooper B , Hardy J , Vassar R , Winblad B , Saido TC (2017) APP mouse models for Alzheimer’s disease preclinical studies. EMBO J 36, 2473–2487. 28768718 10.15252/embj.201797397 PMC5579350

[52] Cacabelos R , Carrera I , Martínez-Iglesias O , Cacabelos N , Naidoo V (2021) What is the gold standard model for Alzheimer’s disease drug discovery and development? Expert Opin Drug Discov 25, 1–26. 10.1080/17460441.2021.1960502 34330186

[53] Myers A , McGonigle P (2019) Overview of transgenic mouse models for Alzheimer’s disease. Curr Protoc Neurosci 89, e81. 31532917 10.1002/cpns.81

[54] Kiyota T , Okuyama S , Swan RJ , Jacobsen MT , Gendelman HE , Ikezu T (2010) CNS expression of anti-inflammatory cytokine interleukin-4 attenuates Alzheimer’s disease-like pathogenesis in APP+PS1 bigenic mice. FASEB J 24, 3093–102. 20371618 10.1096/fj.10-155317 PMC2909296

[55] Kiyota T , Ingraham KL , Swan RJ , Jacobsen MT , Andrews SJ , Ikezu T (2012) AAV serotype 2/1-mediated gene delivery of anti-inflammatory interleukin-10 enhances neurogenesis and cognitive function in APP+PS1 mice. Gene Ther 19, 724–733. 21918553 10.1038/gt.2011.126 PMC3241853

[56] Latta CH , Sudduth TL , Weekman EM , Brothers HM , Abner EL , Popa GJ , Mendenhall MD , Gonzalez-Oregon F , Braun K , Wilcock DM (2015) Determining the role of IL-4 induced neuroinflammation in microglial activity and amyloid-β using BV2 microglial cells and APP/PS1 transgenic mice. J Neuroinflammation 12, 41. 25885682 10.1186/s12974-015-0243-6 PMC4350455

[57] Chakrabarty P , Tianbai L , Herring A , Ceballos-Diaz C , Das P , Golde TE (2012) Hippocampal expression of murine IL-4 results in exacerbation of amyloid deposition. Mol Neurodegener 7, 36. 22838967 10.1186/1750-1326-7-36 PMC3441281

[58] Chakrabarty P , Li A , Ceballos-Diaz C , Eddy JA , Funk CC , Moore B , DiNunno N , Rosario AM , Cruz PE , Verbeeck C , Sacino A , Nix S , Janus C , Price ND , Das P , Golde TE (2015) IL-10 alters immunoproteostasis in APP mice, increasing plaque burden and worsening cognitive behavior. Neuron 85, 519–533. 25619653 10.1016/j.neuron.2014.11.020 PMC4320003

[59] Guillot-Sestier MV , Doty KR , Town T (2015) Innate immunity fights Alzheimer’s disease. Trends Neurosci 38, 674–681. 26549882 10.1016/j.tins.2015.08.008 PMC4641041

[60] Lentz TB , Gray SJ , Samulski RJ (2012) Viral vectors for gene delivery to the central nervous system. Neurobiol Dis 48, 179–88. 22001604 10.1016/j.nbd.2011.09.014 PMC3293995

[61] Abbas N , Bednar I , Mix E , Marie S , Paterson D , Ljungberg A , Morris C , Winblad B , Nordberg A , Zhu J (2002) Up-regulation of the inflammatory cytokines IFN-gamma and IL-12 and down-regulation of IL-4 in cerebral cortex regions of APP(SWE) transgenic mice. J Neuroimmunol 126, 50–57. 12020956 10.1016/s0165-5728(02)00050-4

[62] Michaud JP , Rivest S (2015) Anti-inflammatory signaling in microglia exacerbates Alzheimer’s disease-related pathology. Neuron 85, 450–452. 25654250 10.1016/j.neuron.2015.01.021

[63] Sarlus H , Heneka MT (2017) Microglia in Alzheimer’s disease. J Clin Invest 127, 3240–3249. 28862638 10.1172/JCI90606 PMC5669553

[64] Raikwar SP , Thangavel R , Dubova I , Ahmed ME , Selvakumar PG , Kempuraj D , Zaheer S , Iyer S , Zaheer A (2018) Neuro-immuno-gene- and genome-editing-therapy for Alzheimer’s disease: Are we there yet? J Alzheimers Dis 65, 321–344. 30040732 10.3233/JAD-180422 PMC6130335

[65] Hampel H , Caraci F , Cuello AC , Caruso G , Nisticò R , Corbo M , Baldacci F , Toschi N , Garaci F , Chiesa PA , Verdooner SR , Akman-Anderson L , Hernández F , Ávila J , Emanuele E , Valenzuela PL , Lucía A , Watling M , Imbimbo BP , Vergallo A , Lista S (2020) A path toward precision medicine forneuroinflammatory mechanisms in Alzheimer’s disease. FrontImmunol 11, 456. 10.3389/fimmu.2020.00456 PMC7137904 32296418

[66] Chen JH , Ke KF , Lu JH , Qiu YH , Peng YP (2015) Protection of TGF-β1 against neuroinflammation and neurodegeneration in Aβ1-42-induced Alzheimer’s disease model rats. PLoS One 10, e0116549. 25658940 10.1371/journal.pone.0116549 PMC4319949

[67] Komai T , Inoue M , Okamura T , Morita K , Iwasaki Y , Sumitomo S , Shoda H , Yamamoto K , Fujio K (2018) Transforming growth factor-β and interleukin-10 synergistically regulate humoral immunity *via* modulating metabolic signals. Front Immunol 9, 1364. 29963056 10.3389/fimmu.2018.01364 PMC6010538

[68] Kudrna JJ , Ugen KE (2015) Gene-based vaccines and immunotherapeutic strategies against neurodegenerative diseases: Potential utility and limitations. Hum Vaccin Immunother 11, 1921–1926. 26125436 10.1080/21645515.2015.1065364 PMC4635842

[69] Murlidharan G , Sakamoto K , Rao L , Corriher T , Wang D , Gao G , Sullivan P , Asokan A (2016) CNS-restricted transduction and CRISPR/Cas9-mediated gene deletion with an engineered AAV vector. Mol Ther Nucleic Acids 5, e338. 27434683 10.1038/mtna.2016.49 PMC5330941

[70] Park H , Oh J , Shim G , Cho B , Chang Y , Kim S , Baek S , Kim H , Shin J , Choi H , Yoo J , Kim J , Jun W , Lee M , Lengner CJ , Oh YK , Kim J (2019) *In vivo* neuronal gene editing via CRISPR-Cas9 amphiphilic nanocomplexes alleviates deficits in mouse models of Alzheimer’s disease. Nat Neurosci 22, 524–528. 30858603 10.1038/s41593-019-0352-0

[71] Bartee E , McFadden G (2013) Cytokine synergy: An underappreciated contributor to innate anti-viral immunity. Cytokine 63, 237–240. 23693158 10.1016/j.cyto.2013.04.036 PMC3748162

[72] Ko KS , Lee M , Koh JJ , Kim SW (2001) Combined administration of plasmids encoding IL-4 and IL-10 prevents the development of autoimmune diabetes in nonobese diabetic mice. Mol Ther 4, 313–316. 11592833 10.1006/mthe.2001.0459

[73] Eijkelkamp N , Steen-Louws C , Hartgring SA , Willemen HL , Prado J , Lafeber FP , Heijnen CJ , Hack CE , van Roon JA , Kavelaars A (2016) IL4-10 fusion protein is a novel drug to treat persistent inflammatory pain. J Neurosci 36, 7353–7363. 27413147 10.1523/JNEUROSCI.0092-16.2016 PMC4945660

[74] Steen-Louws C , Hartgring SAY , Popov-Celeketic J , Lopes AP , de Smet MBM , Eijkelkamp N , Lafeber FPJG , Hack CE , van Roon JAG (2019) IL4-10 fusion protein: A novel immunoregulatory drug combining activities of interleukin 4 and interleukin 10. Clin Exp Immunol 195, 1–9. 30307604 10.1111/cei.13224 PMC6300648

[75] Li W , Huang E , Gao S (2017) Type 1 diabetes mellitus and cognitive impairments: A systematic review. J Alzheimers Dis 57, 29–36. 28222533 10.3233/JAD-161250

[76] Biessels GJ , Despa F (2018) Cognitive decline and dementia in diabetes mellitus: Mechanisms and clinical implications. Nat Rev Endocrinol 14, 591–604. 30022099 10.1038/s41574-018-0048-7 PMC6397437

[77] Newcombe EA , Camats-Perna J , Silva ML , Valmas N , Huat TJ , Medeiros R (2018) Inflammation: The link between comorbidities, genetics, and Alzheimer’s disease. J Neuroinflammation 15, 276. 30249283 10.1186/s12974-018-1313-3 PMC6154824

[78] Rea IM , Gibson DS , McGilligan V , McNerlan SE , Alexander HD , Ross OA (2018) Age and age-related diseases: Role of inflammation triggers and cytokines. Front Immunol 9, 586. 29686666 10.3389/fimmu.2018.00586 PMC5900450

[79] Cao S , Fisher DW , Yu T , Dong H (2019) The link between chronic pain and Alzheimer’s disease. J Neuroinflammation 16, 204. 31694670 10.1186/s12974-019-1608-z PMC6836339

[80] King DL , Arendash GW (2002) Behavioral characterization of the Tg2576 transgenic model of Alzheimer’s disease through 19 months. Physiol Behav 75, 627–642. 12020728 10.1016/s0031-9384(02)00639-x

[81] Wang J , Tanila H , Puoliväli J , Kadish I , van Groen T (2003) Gender differences in the amount and deposition of amyloidbeta in APPswe and PS1 double transgenic mice. Neurobiol Dis 14, 318–327. 14678749 10.1016/j.nbd.2003.08.009

[82] Shimazawa M , Inokuchi Y , Okuno T , Nakajima Y , Sakaguchi G , Kato A , Oku H , Sugiyama T , Kudo T , Ikeda T , Takeda M , Hara H (2008) Reduced retinal function in amyloid precursor protein-over-expressing transgenic mice via attenuating glutamate-N-methyl-d-aspartate receptor signaling. J Neurochem 107s, 279–290. 10.1111/j.1471-4159.2008.05606.x 18691390

[83] Bedrosian TA , Herring KL , Weil ZM , Nelson RJ (2011) Altered temporal patterns of anxiety in aged and amyloid precursor protein (APP) transgenic mice. Proc Natl Acad Sci U S A 108, 11686–11691. 21709248 10.1073/pnas.1103098108 PMC3136261

[84] Puzzo D , Gulisano W , Palmeri A , Arancio O (2015) Rodent models for Alzheimer’s disease drug discovery. Expert Opin Drug Discov 10, 703–711. 25927677 10.1517/17460441.2015.1041913 PMC4592281

[85] Esquerda-Canals G , Montoliu-Gaya L , Güell-Bosch J , Villegas S (2017) Mouse models of Alzheimer’s disease. J Alzheimers Dis 57, 1171–1183. 28304309 10.3233/JAD-170045

[86] Sparks DL (2008) The early and ongoing experience with the cholesterol-fed rabbit as a model of Alzheimer’s disease: The old, the new and the pilot. J Alzheimers Dis 15, 641–656. 19096162 10.3233/jad-2008-15410

[87] Woodruff-Pak DS (2008) Animal models of Alzheimer’s disease: Therapeutic implications. J Alzheimers Dis 15, 507–521. 19096153 10.3233/jad-2008-15401

[88] Sims-Robinson C , Kim B , Rosko A , Feldman EL (2010) How does diabetes accelerate Alzheimer disease pathology? Nat Rev Neurol 6, 551–559. 20842183 10.1038/nrneurol.2010.130 PMC3199576

[89] Marciani DJ (2016) A retrospective analysis of Alzheimer’s disease vaccine progress - The critical need for new development strategies. J Neurochem 137, 687–700. 26990863 10.1111/jnc.13608

[90] Lambracht-Washington D , Fu M , Wight-Carter M , Riegel M , Rosenberg RN (2017) Evaluation of a DNA Aβ42 vaccine in aged NZW rabbits: Antibody kinetics and immune profile after intradermal immunization with full-length DNA Aβ42 trimer. J Alzheimers Dis 57, 97–112. 28222511 10.3233/JAD-160947 PMC5345648

[91] Wolff JA , Budker V (2005) The mechanism of naked DNA uptake and expression. Adv Genet 54, 3–20. 16096005 10.1016/S0065-2660(05)54001-X

[92] Jayant RD , Sosa D , Kaushik A , Atluri V , Vashist A , Tomitaka A , Nair M (2016) Current status of non-viral gene therapy for CNS disorders. Expert Opin Drug Deliv 13, 1433–1445. 27249310 10.1080/17425247.2016.1188802 PMC5480312

[93] Kumar SR , Markusic DM , Biswas M , High KA , Herzog RW (2016) Clinical development of gene therapy: Results and lessons from recent successes. Mol Ther Methods Clin Dev 3, 16034. 27257611 10.1038/mtm.2016.34 PMC4879992

